# The monthly spectrum: Premenstrual symptoms across ADHD and autism

**DOI:** 10.1177/17455057261476912

**Published:** 2026-08-19

**Authors:** Annabeth P. Groenman, Lotte E. Welling, Sara Pieters

**Affiliations:** 1 Department of Child and Adolescent Psychiatry Groningen, University Medical Center Groningen, 534843 University of Groningen, Groningen, the Netherlands; 2 Accare Child Study Center, Groningen, the Netherlands; 3 The Research School of Behavioral and Cognitive Neuroscience, 3647 University of Groningen, Groningen, the Netherlands; 4 Research Institute of Child Development and Education, 1234 University of Amsterdam, Amsterdam, the Netherlands; 5 Behavioural Science Institute, 404818 Radboud University, Nijmegen, The Netherlands; 6 Karakter Child and Adolescent Psychiatry, Karakter Academy, Nijmegen, The Netherlands

**Keywords:** premenstrual complaints, ADHD, autism, neurodiversity

## Abstract

**Background:**

Women with ADHD and autism experience elevated rates of premenstrual problems, yet no direct comparisons between these conditions exist in adults.

**Objectives:**

This study examined premenstrual dysphoric disorder (PMDD) prevalence, symptoms, and impairment across ADHD, autism, and neurotypical women.

**Design:**

Cross-sectional data from 199 women aged 20-40 without hormonal contraceptive use were analyzed across two samples.

**Methods:**

Participants included 89 with ADHD, 39 with autism, and 71 controls. Premenstrual symptoms were assessed using the Premenstrual Symptoms Screening Tool (PSST), alongside measures of ADHD characteristics, autism traits, and sensory hypersensitivity.

**Results:**

Both ADHD and autistic women showed significantly elevated provisional PMDD rates according to the PSST screening instrument compared to controls, with no significant difference between neurodivergent groups. Dimensional analyses revealed positive associations between premenstrual symptoms and neurodiverse traits, particularly inattention, hyperactivity-impulsivity, and social skills difficulties. The impact of premenstrual problems was similarly associated with these traits across diagnostic groups. Contrary to hypotheses, sensory hypersensitivity was not independently associated with premenstrual symptoms after controlling for ADHD and autism characteristics.

**Conclusions:**

Premenstrual problems constitute a significant burden for both ADHD and autistic women, with dimensional associations suggesting that individuals with more severe neurodivergent traits face heightened risk. Yet our findings also demonstrate that prevalence estimates are only as reliable as the recruitment strategies behind them. Advancing this field requires both greater clinical attention to menstrual cycle-related difficulties in neurodivergent populations and recruitment strategies that yield dependable estimates.

## Plain Language Summary

Many individuals with ADHD or autistic individuals report premenstrual problems such as low mood, irritability, or headaches in the days before their period. Until now, no study had directly compared these individuals with ADHD or autism to each other, so it was unclear whether one group was more affected than the other. We studied 199 individuals with a menstrual cycle aged 20 to 40 who were not using hormonal contraception: 89 with ADHD, 39 with autism, and 71 individuals without either diagnosis. Everyone completed a short questionnaire about premenstrual symptoms, along with measures of ADHD characteristics and autism traits. A subsample also completed a measure on sensory sensitivity. Individuals with ADHD and autistic individuals were both more likely than those without these classifications to screen positive for premenstrual dysphoric disorder (PMDD), a severe form of premenstrual difficulty. The two neurodivergent groups did not differ from each other. We also found that the more neurodivergent traits a person reported, especially inattention, hyperactivity and impulsivity, and social difficulties, the more premenstrual symptoms she tended to report, and the more those symptoms affected her daily life. This pattern held regardless of classification. Sensory sensitivity was not related to premenstrual problems. Overall, premenstrual problems appear to be a real and meaningful burden for both ADHD and autistic women, and individuals who reported more traits seem to be at greater risk. However, our results carry a note of caution: Our results show a strong indication of selection bias, where those individuals recruited for a study on premenstrual problems showed a much higher prevalence than those recruited for a study on sleep. Progress in this field will require both more clinical attention to menstrual cycle difficulties in neurodivergent women and recruitment methods that produce trustworthy estimates.

## Introduction

Attention deficit/hyperactivity disorder (ADHD), and Autism Spectrum Disorder (from here on autism) have long been overlooked in women [In the current manuscript we include every individual who menstruates, regardless of gender. We will use the term women to indicate those who were assigned female at birth]. The research field has long been dominated by studies on males.^1,2^ Recent years have shown a change towards research in neurodiverse women, and while this line of research is still in its infancy, it has revealed a concerning pattern of problems related to hormonal sensitivity in these women.^3–8^ Increasing quantitative evidence points towards an increased rate of premenstrual problems in those with ADHD, however, in the autism field the quantitative data is less consistent in its pattern, while qualitative data does seem to point to an increase in Premenstrual Dysphoric Disorder (PMDD) in autistic women. Moreover, only one study was able to perform a direct comparison between girls with ADHD and autistic girls,^6^ but until now, the direct comparison between *adults* with ADHD or autism has not been possible. Furthermore, given the high comorbidity between ADHD and autism,^9^ it remains unclear whether premenstrual symptoms are uniquely associated with one condition, the other, or both independently. Without direct comparison, findings from single-condition studies cannot distinguish condition-specific patterns from effects driven by undiagnosed comorbidity. Therefore, this study will compare whether ADHD or autism are uniquely related to premenstrual dysphoric disorder, and whether they show a unique pattern of symptoms.

Premenstrual syndrome is defined as the experience of one or more somatic (i.e. breast tenderness, bloating, joint or muscle pain or weight gain) and/or affective (i.e. marked affective liability, irritability or anger, depressed mood or anxiety) symptoms in the period before menstruation onset, that disappear after the onset of menstruation impairing work, social or school performance.^10^ Around 80% of women fulfill one of the criteria of PMS without the impairment criteria, and around 20-30% also meet an impairment criterium.^11,12^ PMDD is a more severe form of PMS, and according to the DSM-V-TR^13^ requires meeting at least 5 symptoms and functional impairment for the diagnosis. In the general population the prevalence of PMDD is between 1.2 -6.4%.^12^ The field of PMDD is plagued by differential application of diagnostic criteria, and therefore, there are large differences in prevalence rated that are reported. Thus, it is important to not only consider diagnosis, as comparing diagnosis rates across studies can lead to an unclear picture due to varying definitions, but also investigate separate symptoms and impairment criteria to fully understand the pattern of premenstrual problems in neurodiverse women.

In women with a clinical ADHD diagnosis, PMDD rates are elevated between 31.4%- 45.5%.^3,4,6^ The reverse was also found; in women with a PMDD diagnosis, ADHD was more prevalent.^14^ A recent paper found that in those without a clinical diagnosis, but meeting ADHD criteria on a questionnaire, the ratings of PMDD were higher than in those with a clinical ADHD diagnosis.^3^ This might suggest that there is a relation between ADHD characteristics and PMDD symptoms. While findings are rather consistent in the field of ADHD, this is not the case in the autism field. A large qualitative study indicates that autistic women show an increase in both autistic characteristics around their menstruation, as well as an increase in mood problems. Two quantitative studies indeed indicate an increase in PMDD prevalence in autistic women,^15,16^ however, two other studies do not find this pattern.^5,6^ To this date, only one study performed a direct comparison between girls (aged 10-19) with ADHD, ADHD and autism and found that PMDD rates were only increased in ADHD and AuDHD, but not in those who only had an autism diagnosis, in adolescents between 10 and 19 year old.^6^ This suggests that ADHD might be associated with an increase in PMDD rates, but not autism. However, we currently do not know whether this pattern also exist in adults with either ADHD or autism.

One possible explanation for the hypothesized increased risk of PMDD among individuals with ADHD or autism centers on heightened sensory sensitivity. Individuals with ADHD and autistic individuals are more likely to experience increased sensitivity to external stimuli,^17,18^ and they may also exhibit heightened interoceptive sensitivity (i.e., greater awareness of internal bodily sensations). Additionally, autistic individuals report increased sensory sensitivity during the premenstrual phase,^8^ possibly indicating a premenstrual exacerbation of pre-existing problems. Potentially, premenstrual exacerbation of already elevated sensory sensitivity may represent a potential mechanistic pathway linking neurodiversity and PMDD: heightened sensitivity increases awareness of bodily signals, which in turn may elevate PMDD risk.

All in all, the current study will focus on the direct comparison of PMDD rates, symptoms and impairment in women with ADHD, autism or without ADHD or autism. Moreover, we will look whether individual characteristics of ADHD or autism are related to PMDD symptoms and explore whether sensory hypersensitivity is related to PMDD as a potential pathway towards PMDD. The current study has three aims. The first aim is to explore the prevalence of premenstrual problems, PMS and PMDD, in neurodiverse women (those with ADHD or autism) and neurotypical women (those without ADHD or autism). We will also test whether premenstrual problems predict group membership (ADHD, autism, controls), while controlling for relevant covariates. The second aim is to investigate the association between premenstrual problems and neurodiverse traits, including ADHD characteristics and autism characteristics. The third aim is to explore the relationship between sensory hypersensitivity and premenstrual problems in a subsample of women.

## Methods

‘The reporting of this study conforms to the STROBE statement for case control studies (see Supplement for STROBE checklist) (von Elm et al., 2008).

### Participants

The data for this cross-sectional questionnaire-based study were collected in two separate samples. Recruitment for these studies was part of (under)graduate thesis projects in the Netherlands. Sample A was collected at the University of Amsterdam by authors LW and APG, with the purpose of examining PMDD symptoms in women with ADHD and autistic women, and Sample B was collected at the Radboud university Nijmegen as an add-on to an undergraduate thesis project on sleep. For both samples, individuals who met the following exclusion criteria were applied a) no menstrual cycle, b) insufficient understanding of the Dutch language to fill out questionnaires, c) use of hormonal contraceptives D) current pregnancy e) below 20 or above the age of 40. For sample A additional exclusion criteria were being 3 months postpartum, a history of neurological disorders (i.e. epilepsy, stroke, and multiple sclerosis), schizophrenia or having experienced more than one psychotic episode. Participants indicated whether they had a diagnosis of ADHD, autism, or neither.

Sample A contained 165 participants: 87 individuals with ADHD, 38 autistic individuals and 38 individuals without a diagnosis of ADHD or autism aged between 20 and 40-years (*M*_age_ = 32.56, *SD*_age_ = 5.13). Sample B contained 34 participants: 2 individuals with ADHD, 1 individual with autism and 31 individuals without autism or ADHD (*M*_age_ = 23.96, *SD*_age_ = 4.76).

### Study design and procedure

Data collection of Sample A took place between February and April 2024. Participants were recruited via advertisements through a Dutch Autism network (the Female Autism Network of the Netherland https://www.fann-autisme.nl/), a Dutch network for individuals with ADHD or dyslexia (Impuls & Woortblind, https://www.impulsenwoortblind.nl/), social media (Facebook and LinkedIn) and via the social networks of the researchers. The research advertisement stated the aim and the procedure of the research and the exclusion criteria. Participants got the possibility to enter a lottery for one of three vouchers of 10 euros. The study set up to collect sample A was approved by the Ethics Review Board of the University of Amsterdam on February 11th, 2024 (FMG-4400).

Data from sample B were collected between April and June 2025. Participants from the general population were recruited via the social networks of the researchers and social media. This study was approved by the Ethics Committee of the Faculty of Social Sciences of Radboud University on April 3^d^ 2025 (ECSW-2025-052).

All participants provided written informed consent prior to participation.

### Measures

#### Premenstrual Symptoms Screening Tool (PSST)

The Dutch translation of the PSST has been used to determine the self-reported PMDD symptoms of the participants.^19^ The PSST is a retrospective tool which includes a list of 14 premenstrual symptoms as well as a measure of impairment in accordance with DSM-IV criteria for PMDD. First, participants were asked to rate their experience of 14 PMDD symptoms (e.g., depressed mood and feeling overwhelmed) on a 4-point Likert scale in the week before the onset of their menstruation and disappearing again within a few days of bleeding. The impairment domain assessed impairment in 5 domains (i.e. productivity, relationships with colleagues, relationships with family, social activities, and domestic chores). Two sum score were computed by adding the scores of all items, one for symptoms and one for the impact of premenstrual symptoms, with a higher score indicating more severe PMDD symptoms. Although specific research on the psychometric qualities of the Dutch version has not yet been conducted, studies in other languages have demonstrated sufficient reliability and validity for the PSST.^20,21^ Cronbach’s alpha in the current sample was .90 for the symptoms subscale and .88 for the impact subscale, indicating good internal consistency reliability. It is important to note that the PSST is considered a screening tool and cannot be used to diagnose PMDD. In this study, no information on clinical diagnosis of PMDD were available. The developers of the PSST have determined that a symptom may be considered present when it is scored as a 3 or higher.^22^ According to the scoring guidelines, a provisional diagnosis of PMDD is made when: (1) at least one of the four core symptoms (items 1–4) is rated as ‘severe’; (2) at least four of the fourteen symptom items are rated as ‘moderate’ or ‘severe’; and (3) at least one of the five functional impairment items (A–E) is rated as ‘severe’. Moderate-to-severe PMS is indicated when: (1) at least one of the core symptoms (items 1–4) is rated as ‘moderate’ to ‘severe’; (2) at least four symptom items (1–14) are rated ‘moderate’ to ‘severe’; and (3) at least one functional impairment item (A–E) is rated ‘moderate’ to ‘severe’.

#### The dutch Glasgow Sensory Questionnaire (GSQ-NL)

The GSQ-NL is a 42-item sensory sensitivity self-report questionnaire, which has been used to measure the participants’ sensory sensitivity (Kuiper et al., 2019). The GSQ-NL is only collected in sample A. Each item refers to one’s behavior to certain sensory stimuli or sensory preferences. The questionnaire consists of seven modalities (i.e. visual, auditory, gustatory, olfactory, tactile, vestibular, and proprioception). Each modality contains six items, three hyposensitivity items (e.g. ‘Do you really like listening to certain sounds?’) and three hypersensitivity items (e.g. ‘Do you dislike loud noises?’). Participants had to answer the questions by a 5-point Likert scale. For this study only the 21 hypersensitivity items were administered. A sum score was computed by adding the scores on all 21 hypersensitivity items, with a higher score indicating higher hypersensitivity. Research shows that the GSQ-NL contains a sufficient internal consistency, convergent and divergent validity and test-retest reliability.^23^ Cronbach’s alpha in the current sample was .91, indicating good internal consistency reliability.

#### Autism-spectrum Quotient-short (AQ-28)

The Dutch version of the AQ-28 was administered (Hoekstra et al., 2008). This survey measures autism traits and comprises 28 statements (e.g., “I find social situations easy”). Participants had to indicate their level of agreement with each statement using a Likert-type scale consisting of 4 levels. Reverse coding was applied to 14 items, which had to be accounted for when summing the items to create a total score. Furthermore, sum scores for four different subscales were calculated. These subscales index distinct dimensions of autistic traits: Routine assesses preference for sameness and resistance to change; Switching reflects cognitive flexibility; Numbers/Patterns captures heightened interest in numerical and patterned information; and Social Skills evaluates social communicative functioning. A higher score on the AQ-short subscales and total score indicates a greater presence of autism characteristics. The Dutch version of the AQ-28 has proven to be reliable for assessing autistic traits, with an internal consistency coefficient measured using Cronbach’s alpha ranging between 0.77 and 0.86.^24^ Cronbach’s alpha of the total score in the current sample was .81, indicating good internal consistency reliability.

#### ADHD-Rating Scale (ADHD-RS)

The Dutch version of the ADHD Rating Scale for Adults (ADHD-RS)^25^ was used to measure ADHD characteristics. The ADHD-RS is a 23-item self-report measure assessing the frequency of DSM-based ADHD characteristics. For the current study, we used the self-report items for adulthood. Items are rated on a 4-point Likert scale (0 = never/rarely to 3 = very often) yielding a total score where higher scores indicate more ADHD characteristics. The scale comprises two theoretically derived subscales that correspond to the core symptom domains of ADHD: Inattention and Hyperactivity/Impulsivity. The Inattention subscale captures difficulties in sustaining attention, organizing tasks, and regulating mental effort, whereas the Hyperactivity/Impulsivity subscale reflects heightened motor activity, restlessness, and difficulties with inhibitory control. Cronbachs’s alpha of the Inattention (α = .87) and Hyperactivity/Impulsivity (α = .82) subscales suggested good internal consistency reliability.

### Statistical analyses

All analyses were conducted using IBM SPSS Statistics (Version 29).^26^ Only available data were analyzed. Missing data ranged between 0 and 5%, for an overview per variable see Supplement S4. Descriptive statistics for all quantitative study variables were calculated and stratified by diagnostic group (ADHD, autism, controls). Pearson’s correlations were then computed between all quantitative study variables. An a priori power analysis for a two-sided test of proportions (α = .05, power = .80), using conservative prevalence estimates of 6.4% for controls and 31.4% for ADHD, indicated a minimum of 34 participants per group. Due to overwhelming enthusiasm for the study, especially in the ADHD group, we ended up with a higher number of participants, for which we later matched the numbers in an additional control group (Sample B).

To examine whether premenstrual problems and their impact predict diagnostic group membership, several multinomial logistic regression analyses were performed, controlling for age. In the first model, premenstrual problems were entered as the predictor, with diagnostic group as the outcome variable and age as a covariate. In the second model, premenstrual impact was entered as the predictor, with diagnostic group as the outcome and age as a covariate.

To further investigate the association between premenstrual problems and their impact on neurodiverse traits, Multivariate Analyses of Covariance (MANCOVA) were conducted. This model contained premenstrual problems or premenstrual impact, diagnostic group, and the interaction between these two as the independent variables, age as a covariate, and subscales of neurodiverse traits (hyperactive/impulsivity (ADHD-RS), inattention (ADHD-RS), routine (AQ), switching (AQ), numbers/patterns (AQ), social skills (AQ), imagination (AQ) as dependent variables.

Finally, the potential relationships between sensory hypersensitivity and premenstrual problems and their impact was explored using two Analyses of Covariance (Ancova). In the first model, sensory hypersensitivity, diagnosis, and their interaction were entered as the predictors, and premenstrual problems as the outcome variable. Age was included as a covariate. In the second model, premenstrual impact was the outcome variable. Both models were repeated, controlling for autistic and ADHD traits. These analyses were conducted only in a subsample of participants (Sample A), as the sensory hypersensitivity questionnaire was administered exclusively in that sample.

## Results

### Descriptive statistics and correlations

Means and standard deviations of all study variables are shown in Table 1, stratified by diagnostic group. With respect to ADHD characteristics, results from Analyses of Variance (ANOVAs) and follow-up post-hoc (Tukey) analyses, consistently showed that the ADHD group, on average, reported more ADHD characteristics compared to the autism (*p* < .001) and control (*p* < .001) groups. Likewise, on nearly all autism-related outcomes, the autism group reported more autism characteristics than the ADHD (*p* < .001) and control groups (*p* < .001). However, for the switching subscale of the AQ, which examines cognitive flexibility, all pairwise comparisons were significant (*p* < .001). Results indicated that the control group was, on average, younger than the autism (*p* = .024) and ADHD groups (*p* = .021). Regarding sensory hypersensitivity, which was assessed only in sample A, the results indicated significant differences between all groups (autism vs. ADHD: *p* = .013; autism vs. controls: *p* < .001; ADHD vs. controls: *p* = .006).

**Table 1.** Descriptive statistics.

​	Autism (n = 39) M (SD)	ADHD (n = 89) M (SD)	Controls (n = 71) M (SD)	Tests of group differences F, p
Age	32.64 (.93)	32.07 (.61)	29.59 (.69)	*F*(2, 196) = 4.91, *p* = .008
Attention problems (ADHD-rs)	9.76 (.86)	18.95 (.57)	7.81 (.63)	*F*(2, 186) = 95.37, *p* < .001
Hyperactivity-impulsivity (ADHD-rs)	13.11 (.96)	21.10 (.64)	10.43 (.71)	*F*(2, 186) = 67.79, *p* < .001
Routine (AQ)	13.41 (.35)	10.05 (.24)	9.89 (.27)	*F*(2, 189) = 37.46, *p* < .001
Switching (AQ)	13.36 (.36)	11.45 (.25)	9.46 (.28)	*F*(2, 189) = 38.01, *p* < .001
Numbers / patterns (AQ)	13.15 (.57)	9.75 (.39)	9.38 (.43)	*F*(2, 189) = 15.72, *p* < .001
Social skills (AQ)	21.87 (.61)	15.27 (.41)	14.82 (.46)	*F*(2, 189) = 50.47, *p* < .001
Imagination (AQ)	19.44 (.59)	13.15 (.40)	13.90 (.45)	*F*(2, 189) = 41.11, *p* < .001
Autism (AQ, total score)	81.23 (1.61)	59.67 (1.09)	57.44 (1.19)	*F*(2, 189) = 79.86, *p* < .001
ADHD (ADHD-rs, total score)	22.87 (1.65)	40.05 (1.09)	18.24 (1.22)	*F*(2, 186) = 96.71, *p* < .001
Sensory hypersensitivity (GSQ; only sample A)	62.40 (2.21)	54.77 (1.48)	46.56 (2.18)	*F*(2, 159) = 13.05, *p* < .001
Premenstrual problems	29.67 (1.33)	30.94 (.88)	23.07 (.98)	*F*(2, 196) = 18.96, *p* < .001
Impact of premenstrual problems	9.51 (.63)	8.92 (.42)	6.11 (.47)	*F*(2, 196) = 13.50, *p* < .001

A series of χ^2^ tests were conducted to examine associations between provisional diagnoses of PMS and PMDD based on the PSST, and diagnostic group (autism, ADHD, controls). Results showed an association between diagnostic group and provisional PMDD diagnosis (χ^2^ (2) = 17.95, *p* < .001), with autistic individuals (61.5%) and individuals with ADHD (66.3%) reporting higher rates of PMDD diagnosis compared to controls (33.8%). In addition, an association was found between diagnostic group and provisional PMS diagnosis (χ^2^ (2) = 20.25, *p* < .001). Percentages indicated a higher prevalence of PMS diagnosis in autistic individuals (92.3%) and individuals with ADHD (88.9%) compared to controls (64.8%).

Pearson’s correlations between all variables are displayed in Table S1. We found correlations >.8 within scales for premenstrual symptoms and impact, and between the total AQ score and imagination. Therefore, these were never entered in the same models.

### The association between premenstrual problems, their impact, and diagnostic group (ADHD, autism, control group)

Several multinomial logistic regression analyses were performed to explore the association between premenstrual problems and their impact on diagnosis (ADHD, autism, or control group). Results are presented in Table 2. The first multinomial logistic regression analysis tested if premenstrual problems, corrected for age, predicted diagnostic group membership (autism, ADHD, control group; latter as reference category). The model with predictors differed significantly from the null model (χ^*2*^ (4) = 34.65, *p* < .001) and explained 18% of the variance (Nagelkerke *R*^*2*^ = .18). Premenstrual problems were significantly associated with autism and ADHD. Specifically, higher levels of premenstrual problems were associated with increased odds of being in the autism or ADHD groups relative to the control group, with a slightly stronger effect observed for ADHD. Using ADHD as a reference group to compare the autism and ADHD groups, we found that PSST symptom score did not significantly differentiate the autism group from ADHD (*B* = -.03, *SE* = .03, *p* = .224, OR = .97, 95% CI [.91 – 1.02]).

**Table 2.** Results of the multinomial regression analyses: The relationship between premenstrual problems and diagnosis.

Predictor	B	SE	Wald χ2	p	OR[95%CI]
*Premenstrual problems*					
**Autism vs. Controls**					
Age	.04	.04	1.10	.294	1.04 [.96 – 1.13]
Premenstrual problems	.07	.03	6.98	.008	1.08 [1.02 – 1.14]
**ADHD vs. Controls**					
Age	.01	.03	.03	.872	1.01 [.94 – 1.07]
Premenstrual problems	.11	.02	20.07	< .001	1.11 [1.06 – 1.17]
**Autism vs. ADHD**					
Age	-.96	1.21	.92	.34	1.04 [.96 – 1.12]
Premenstrual problems	.04	.4	1.48	.22	.97 [.91 – 1.02]
*Impact of premenstrual problems*					
**Autism vs. Controls**					
Age	.03	.04	.65	.421	1.03 [.95 – 1.12]
Impact of premenstrual problems	.20	.06	10.73	.001	1.22 [1.08 - 1.37]
**ADHD vs. Controls**					
Age	.02	.03	.58	.447	1.02 [.96 - 1.09]
Impact of premenstrual problems	.16	.05	11.63	< .001	1.18 [1.07 - 1.29]
**Autism vs. ADHD**					
Age	.01	.04	.05	.820	1.01 [.93 - 1.09]
Impact of premenstrual problems	.03	.06	.38	.536	1.03 [.93 - 1.15]

A second multinomial logistic regression analysis tested if age and the impact of premenstrual problems predicted group membership (autism, ADHD, control group). The model with predictors differed significantly from the null model (χ^2^ (4) = 25.98, *p* < .001) and explained 14% of the variance (Nagelkerke *R*^*2*^ = .14). Greater impact of premenstrual problems significantly increased the odds of being in the autism and ADHD groups, with a slightly stronger effect observed for autism. Using ADHD as a reference group to compare the autism and ADHD groups, we found that the impact of premenstrual problems did not differentiate between the autism and the ADHD group (See Table 2 for full results).

### The association between premenstrual problems, their impact, and neurodiverse traits

The association between premenstrual problems, their impact and neurodiverse traits (all ADHD and autism related subscales) were examined in two MANCOVAs. In the first analysis, the association between age, diagnostic group, premenstrual problems (Z-score) and the interaction between diagnostic group and premenstrual problems, and neurodiverse traits was examined. The interaction between diagnostic group and premenstrual problems was not significant (Wilks’ λ = .92, *F* (14, 352) = 1.06, *p* = .391, *η*^*2*^_*p*_ = .04), indicating that associations between the impact of premenstrual problems and neurodiverse traits were not different between groups. Results did reveal a significant multivariate main effect of premenstrual problems on neurodiverse traits (Wilks’ λ = .85, *F* (7, 176) = 4.62, *p* < .001, *η*^*2*^_*p*_ = .16). Specifically, follow-up tests indicated that premenstrual problems were positively related to all neurodiverse traits, except for numbers/patterns and imagination (AQ; see Table 3).

**Table 3.** Parameter estimates: The association between premenstrual problems and neurodiverse traits.

Outcome	*B (SE)*	*p*
**Attention problems**	**2.13 (.59)**	**< .001**
**Hyperactivity-impulsivity**	**2.60 (.66)**	**< .001**
**Routine**	**.59 (.26)**	**.025**
**Switching**	**.57 (.26)**	**.032**
Numbers/patterns	.35 (.42)	.398
**Social skills**	**1.07 (.44)**	**.016**
Imagination	.24 (.44)	.594

Results also indicated a significant main effect of diagnostic group on the combined dependent variables (Wilks’ λ = .29, *F* (14, 352) = 21.75, *p* < .001, *η*^*2*^_*p*_ = .46). Univariate follow-up tests and estimated marginal means are shown in Table S2. Results indicated that ADHD characteristics were in general higher in the ADHD group compared to the other diagnostic groups, while autism characteristics were in general higher in the autism group compared to the other diagnostic groups.

In the second MANCOVA model premenstrual problems were replaced with the impact of premenstrual problems. The interaction between diagnostic group and premenstrual problems was not significant (Wilks’ λ = .95, *F* (14, 352) = .71, *p* = .760, *η*^*2*^ = .03). There was a significant multivariate main effect of the impact of premenstrual problems on neurodivergent traits (Wilks’ λ = .86, *F* (7, 176) = 4.26, *p* < .001, *η*^*2*^ = .15). Follow-up tests showed that the impact of premenstrual problems was positively associated with attention problems, hyperactivity-impulsivity, and social skills (see Table 4).

**Table 4.** Parameter estimates: The association between the impact of premenstrual problems and neurodiverse traits.

Outcome	*B (SE)*	*p*
**Attention problems**	**1.40 (.63)**	**.028**
**Hyperactivity-impulsivity**	**1.71 (.71)**	**.017**
Routine	.51 (.27)	.063
Switching	.49 (.28)	.080
Numbers/patterns	.69 (.44)	.119
**Social skills**	**1.75 (.45)**	**< .001**
Imagination	.47 (.46)	.311

Results also indicated a significant main effect of diagnostic group on the combined dependent variables (Wilks’ λ = .29, *F* (14, 352) = 21.83, *p* < .001, *η*^*2*^ = .47). Univariate follow-up tests and estimated marginal means are presented in Table S3.

### The association between sensory hypersensitivity and premenstrual problems and their impact (sample A)

Two ANCOVAs were performed to examine the association between sensory hypersensitivity and premenstrual problems and their impact, exclusively in participants of sample A. In the first analysis, diagnostic group (ADHD, autism, and control group) and sensory hypersensitivity and their interaction, were included as independent variables, and premenstrual problems as a dependent variable, while controlling for age. While the overall model containing age, diagnostic group (ADHD, autism, control group) and sensory hypersensitivity showed a significant overall effect on premenstrual symptoms (*F* (1, 155) = 13.62, *p* < .001, *η*^*2*^_*p*_ = .08), the individual coefficient of sensory hypersensitivity, controlling for diagnosis and age, did not reach statistical significance (*B* = 2.24, *SE* = 1.19, *t* = 1.89, *p* = .061, *η*^*2*^_*p*_ = .02), This is likely a reflection that the F-test evaluates the total contribution of sensory sensitivity across the model, whereas the t-test assesses its unique effect after accounting for other covariates. Neither the main effect of diagnosis (*F* (2, 155) = 3.04, *p* = .051, *η*^*2*^_*p*_ = .04) nor the interaction between diagnosis and sensory hypersensitivity (*F* (2, 155) = .06, *p* = .945, *η*^*2*^_*p*_ = .00) were significant. Repeating this analysis while controlling for neurodiverse traits (i.e., total score on ADHD-rs and total score on AQ), the association between sensory hypersensitivity and premenstrual problems was no longer found (*F* (1, 146) = 2.11, *p* = .148, *η*^*2*^_*p*_ = .01).

The association between sensory hypersensitivity and the impact of premenstrual problems was examined in another ANCOVA, including age as a covariate and diagnostic group as a between-subject factor. The overall model was significant in predicting impact of premenstrual problems (*F* (1, 155) = 6.60, *p* = .011, *η*^*2*^_*p*_ = .04). Again, the individual coefficient of sensory hypersensitivity was not significant (*B* = .81, *SE* = .60, *t* = 1.34, *p* = .182, *η*^*2*^_*p*_ = .01). Following the abovementioned analyses for premenstrual symptoms, the ANCOVA was also repeated controlling for ADHD and autism characteristics. Results of this ANCOVA showed that the association between sensory hypersensitivity and the impact of premenstrual problems was not significant (*F* (1, 146) = .18, *p* = .672, *η*^*2*^_*p*_ = .00).

## Discussion

The present study aimed to examine premenstrual problems in neurodiverse women with ADHD or autism compared to neurotypical controls. Specifically, we investigated (1) group differences in the prevalence of PMDD rates based on the PSST screening instrument, PMDD symptoms and experienced impairment (2) associations between premenstrual problems and neurodiverse traits, and (3) the relationship between sensory hypersensitivity and premenstrual difficulties. Overall, the findings demonstrated that women with ADHD and autism reported more severe premenstrual problems and a greater impact on daily functioning compared to neurotypical women, but that there was no significant difference between women with ADHD or autism. Both premenstrual problems and their impact were positively associated with ADHD characteristics and autism traits, particularly difficulties in attention, hyperactivity–impulsivity, and social skills.

Our findings indicate that both the severity and impact of premenstrual symptoms were significantly elevated in women with ADHD or autism classifications, compared to those without these classifications, echoing results of previous studies.^3,4,6^ To our current knowledge, we are the first to show that PMDD risk is elevated both in ADHD and autistic adult women on a self-report measure, with no significant difference between those with an ADHD or autism diagnosis. One point that needs attention, is the overlap between symptoms of PMDD and core characteristics of ADHD and autism. For example, difficulty concentrating is a symptom of PMDD and ADHD. Moreover, individuals with autism and/or ADHD might also experience premenstrual problems uniquely related to their classification, such as autistic individuals reporting increased sensory sensitivity during the premenstrual phase.^8^ These experiences unique to those with ADHD/autism are not captured by current instruments to measure PMDD.

We found remarkable differences in the prevalence estimates of PMS and PMDD rates based on the PSST instrument between the control groups of sample A and sample B (PMS: 92.1% vs. 48.4%; PMDD: 60.5% vs. 3.2%). Moreover, the PMDD rates reported by neurodiverse women in sample A is far higher than previous studies indicate (i.e., 31.4%- 45.5%^3,4,6^) (an overview of differences between all key variables can be found in the supplement). This difference is quite likely the results of recruitment methods causing selection bias, where in sample A we recruited participants with the text “for a study on premenstrual experiences…”, where in sample B participants were recruited with the text “How well do you sleep?”. In sample A we already chose the more neutral “experiences” instead of “complaints”, however, there is strong reason to believe the data suffer from selection bias. This focus on premenstrual experiences might have attracted those individuals that have pronounced premenstrual experiences. Indeed, an experimental manipulation on wording in recruitment text for social media recruiting found that when the text contained “mental health” participants had worse mental health, than when recruitment texts were focused on strengths.^27^ While the difference in recruitment text could be one reason, social media recruitment and its underlying algorithm, leaves open the possibility, that not just the content of the text, but also social media biased algorithms disproportionately target specific groups.^28^ While this difference in prevalence is a strong indication that the prevalence rates of PMDD in the current study are not reliable, it does provide information about the characteristics of individuals suffering from PMDD. When looking at the bigger picture, this finding emphasizes the critical importance of careful participant recruitment and highlights how subtle differences in recruitment language can substantially influence sample composition and prevalence estimates. Future studies should carefully draft recruitment procedures to avoid inflating prevalence rates and remain vigilant about potential selection biases that may affect the generalizability of findings.

While qualitative studies report a premenstrual exacerbation of sensory hypersensitivity in autistic individuals,^8^ we did not find a link between overall sensory hypersensitivity and premenstrual symptoms or their impact. However, we used an overall measure, and not a measure that explicitly asked about premenstrual exacerbation of sensory sensitivity. Therefore, it could potentially be that while trait level sensory hypersensitivity is not related to premenstrual complaints, premenstrual exacerbation of pre-existing hypersensitivity might be. Recent preclinical evidence has linked progesterone receptors to sensory sensitivity in mice,^29^ offering a potential mechanistic pathway connecting premenstrual symptoms to sensory processing. While one pathway explaining all relations is too simplistic and current evidence for this only stems from preclinical research, altered levels or functioning of progesterone receptors could provide a biological explanation for the association between neurodiversity and premenstrual complaints. As autistic individuals report that they experience increased sensory sensitivity, and also premenstrual exacerbation of hypersensitivity,^8^ and because there is a potential biological pathway, future studies on premenstrual experiences in neurodivergent women should include sensory hypersensivity as potential pathway towards premenstrual problems.

Nevertheless, our dimensional results show a link between premenstrual problems and attention problems, hyperactivity–impulsivity, routine, switching and social skills, and between impact of premenstrual problems and attention problems, hyperactivity–impulsivity, and social skills. This pattern has important clinical implications, as it suggests that individuals with more severe ADHD and autistic traits may be at heightened risk for clinically significant premenstrual problems. Moreover, these associations may reflect a bidirectional relationship, where premenstrual hormone fluctuations temporarily intensify neurodevelopmental symptoms, creating a cyclical pattern of symptom exacerbation. While there currently is no research on premenstrual exacerbation of symptoms in groups with ADHD or autism, research in students indicate that at least ADHD symptoms fluctuate over the cycle,^30^ indicating that those individuals that are hormonally sensitive, might also show premenstrual exacerbation of autistic and or ADHD traits. However, the field is currently challenged by unclear diagnostic criteria for PMS, PMDD, and PME of an existing mental health condition, leading to inconsistent assessments.^12^ This indicated a clear need for instruments that can disentangle between exacerbation of pre-existing conditions, and premenstrual symptoms.

### Limitations

The current research has several strengths, including comparisons across classifications and a dimensional next to a categorical approach. However, the results should be interpreted in the light of several limitations. Future research should employ longitudinal rather than cross-sectional designs, as the screening measure used in this study captures symptoms at a single time point. Nevertheless, the substantial group differences observed suggest clinically meaningful elevations in premenstrual symptomatology among neurodiverse women. First, while guidelines include the PSST as potential screening instrument, they also recognize that it is not well suited to map the cyclical nature of PMDD. Previous research even found that 40% of women that indicated they suffered from premenstrual symptoms, did not meet criteria when measures prospectively.^12^ Therefore, it is often recommended to use an instrument like the Daily Record of Severity of Problems (DRSP) to report symptoms over two cycles,^31^ as the PSST is not suitable as an instrument for official diagnosis. Second, we did not measure hyposensitivity while this is often reported by neurodiverse women. We made this choice to reduce the burden on our participants. However, this could be a potential interest as neurodivergent women report both hyper and hypo sensory sensitivity.^32^

## Conclusion

This study demonstrates that premenstrual problems constitute a substantial burden for women with ADHD and autism, with implications for both clinical practice and future research. Greater clinical awareness of menstrual cycle-related difficulties in neurodiverse populations is needed, alongside the development of more sensitive, neurodivergent-informed measures to fully capture the scope and mechanisms of these experiences. Ultimately, our findings underscore that the question of who participates in menstrual health research is inseparable from the question of how they are recruited, as our results seem highly influenced by a selection bias. This important methodological consideration has been largely overlooked in the field and is of the utmost importance to address so that the field can arrive at robust and generalizable prevalence estimates.

## Supplemental material

Supplemental material - The monthly spectrum: Premenstrual symptoms across ADHD and autism Supplemental material for The monthly spectrum: Premenstrual symptoms across ADHD and autism by A.P. Groenman, L.E. Welling, and S. Pieters in Women’s Health.

## Data Availability

Anonymous data can be shared on reasonable request by the corresponding author.*
