# Supplementary material for: The monthly spectrum: Premenstrual symptoms across ADHD and autism
Source: Womens Health (Lond). 2026 Aug 19;22:17455057261476912. doi: 10.1177/17455057261476912 (PMC13662566; doi:10.1177/17455057261476912)
Supplement: Supplementary file 1 [file sj-pdf-1-whe-10.1177_17455057261476912.pdf]

Supplementary Materials

Table of Contents

STROBE Checklist — Case-Control Studies 2

Table S1 Pearson's Correlations 5

Table S2 Univariate follow-up tests examining differences between diagnostic groups and neurodiverse traits 6

Table S3 Univariate follow-up tests examining differences between diagnostic groups and neurodiverse traits 8

Table S4. Overview of Missing Data Across Study Variables 9

Table S5. Means, Standard Deviations, and Independent-Samples *t*-Test Results by University .10

STROBE Checklist — Case-Control Studies

Manuscript: The Monthly Spectrum: Premenstrual Symptoms Across ADHD and Autism

Authors: Groenman, Welling & Pieters

Item No	Section	Recommendation	Reported on page / Comment
**Title and abstract**			
1	*Title and abstract*	(a) Indicate the study's design with a commonly used term in the title or the abstract	Abstract p 2.
		(b) Provide in the abstract an informative and balanced summary of what was done and what was found	Abstract: P. 2.
**Introduction**			
2	*Background / rationale*	Explain the scientific background and rationale for the investigation being reported	Introduction p. 3-4
3	*Objectives*	State specific objectives, including any prespecified hypotheses	Introduction, p.5.
**Methods**			
4	*Study design*	Present key elements of study design early in the paper	Methods, p.6.
5	*Setting*	Describe the setting, locations, and relevant dates, including periods of recruitment, exposure, follow-up, and data collection	Methods, p.6-7.
6	*Participants*	(a) Give the eligibility criteria, and the sources and methods of case ascertainment and control selection. Give the rationale for the choice of cases and controls	Methods, P.6.
		(b) For matched studies, give matching criteria and the number of controls per case	N/A —
7	*Variables*	Clearly define all outcomes, exposures, predictors, potential confounders, and effect modifiers. Give diagnostic criteria, if applicable	Methods, p.7-9.
8	*Data sources /*	For each variable of interest, give sources of data and details of methods of assessment	Methods p.7-9.

	measurement	(measurement). Describe comparability of assessment methods if there is more than one group	
9	*Bias*	Describe any efforts to address potential sources of bias	Discussion: p.17.
10	*Study size*	Explain how the study size was arrived at	Methods, p.6.
11	*Quantitative variables*	Explain how quantitative variables were handled in the analyses. If applicable, describe which groupings were chosen and why	Methods, p.7-10.
12	*Statistical methods*	(a) Describe all statistical methods, including those used to control for confounding	Methods, p. 9-10
		(b) Describe any methods used to examine subgroups and interactions	Methods, p. 9-10
		(c) Explain how missing data were addressed	Methods, p. 9-10
		(d) If applicable, explain how matching of cases and controls was addressed	N/A — no matching.
		(e) Describe any sensitivity analyses	Methods, p.10.
**Results**			
13*	*Participants*	(a) Report numbers of individuals at each stage of study — e.g. numbers potentially eligible, examined for eligibility, confirmed eligible, included in the study, completing follow-up, and analysed	Methods p.6.
		(b) Give reasons for non-participation at each stage	NA, the study did not partake in different stages.
		(c) Consider use of a flow diagram	See 13b
14*	*Descriptive data*	(a) Give characteristics of study participants (e.g. demographic, clinical, social) and information on exposures and potential confounders	Results, p.12.
		(b) Indicate number of participants with missing data for each variable of interest	Methods, p.10.

15*	*Outcome data*	Report numbers in each exposure category, or summary measures of exposure	Results: p.12.
16	*Main results*	(a) Give unadjusted estimates and, if applicable, confounder-adjusted estimates and their precision (e.g., 95% confidence interval). Make clear which confounders were adjusted for and why they were included	Results, p.13-15
		(b) Report category boundaries when continuous variables were categorized	Methods, p.8.
		(c) If relevant, consider translating estimates of relative risk into absolute risk for a meaningful time period	N/A — cross-sectional prevalence study.
17	*Other analyses*	Report other analyses done — e.g. analyses of subgroups and interactions, and sensitivity analyses	Methods, p.11.
**Discussion**			
18	*Key results*	Summarise key results with reference to study objectives	Discussion, paragraph 1: Key findings summarised for all three aims.
19	*Limitations*	Discuss limitations of the study, taking into account sources of potential bias or imprecision. Discuss both direction and magnitude of any potential bias	Discussion, p.16.
20	*Interpretation*	Give a cautious overall interpretation of results considering objectives, limitations, multiplicity of analyses, results from similar studies, and other relevant evidence	Discussion: p,16-18
21	*Generalisability*	Discuss the generalisability (external validity) of the study results	Discussion: p,16-20.
**Other information**			
22	*Funding*	Give the source of funding and the role of the funders for the present study and, if applicable, for the original study on which the present article is based	Declarations: p.20-21

Table S1 Pearson's Correlations

	1	2	3	4	5	6	7	8	9	10	11
1. Age											
2. Premenstrual problems	.45***										
3. Impact of premenstrual problems	.47***	.82***									
4. Attention problems (ADHD-rs)	.16**	.42***	.32***								
5. Hyperactivity-impulsivity (ADHD-rs)	.04	.41***	.29***	.81***							
6. Routine (AQ)	.13	.24***	.27***	-.11	-.04						
7. Switching (AQ)	.24***	.40***	.38***	.33***	.23**	.47***					
8. Numbers/patterns (AQ)	-.02	.15*	.19*	-.01	.15*	.40***	.32***				
9. Social skills (AQ)	.11	.22**	31***	-.08	-.10	.58***	.42***	.46***			
10. Imagination (AQ)	.05	.04	.10	-.12	-.09	.45***	.39***	.30***	.54***		
11. Autism (AQ total score)	.12	.26***	.32***	-.03	.02	.74***	.65***	.68***	.84***	.76***	

12. Sensory hypersensitivity (GSQ; sample A only)	-.05	.30***	.21**	.17*	.31***	.42***	.37***	.55***	.42***	.28***	.55***

Note. * $p < .05$, ** $p < .01$, *** $p < .001$

Table S2 Univariate follow-up tests examining differences between diagnostic groups and neurodiverse traits

Outcome	F(2, 182) =	p	η^2	*ADHD (EMM*, SE**)*	*Autism (EMM, SE)*	*Controls (EMM, SE)*	*Significant pairwise comparisons*
Attention problems	75.84	< .001	.46	19.01 (.61)	9.27 (.85)	8.94 (.68)	ADHD > autism<br>ADHD > controls
Hyperactivity-impulsivity	51.04	< .001	.36	21.02 (.67)	12.86 (.95)	11.52 (.75)	ADHD > autism<br>ADHD > controls
Routine	32.30	< .001	.26	9.85 (.27)	13.39 (.38)	10.20 (.30)	Autism > ADHD<br>Autism > controls
Switching	23.96	< .001	.21	11.27 (.27)	13.13 (.38)	9.82 (.30)	Autism > ADHD<br>Autism > controls<br>ADHD > controls
Numbers/patterns	13.57	< .001	.13	9.49 (.43)	12.99 (60)	9.42 (.48)	Autism > ADHD<br>Autism > controls
Social skills	49.97	< .001	.35	14.92 (.45)	22.22 (.63)	15.31 (.50)	Autism > ADHD<br>Autism > controls
Imagination	34.22	< .001	.27	13.18 (.45)	19.39 (.64)	13.98 (.51)	Autism > ADHD<br>Autism > controls

Note. * EMM = Estimated Marginal Mean, ** SE = Standard Error.

Table S3 Univariate follow-up tests examining differences between diagnostic groups and neurodiverse traits

Outcome	*F*(2, 182) =	*p*	$\acute{\eta}^2$	*ADHD (EMM*, SE**)*	*Autism (EMM, SE)*	*Controls (EMM, SE)*	*Significant pairwise comparisons*
Attention problems	83.62	< .001	.48	18.84 (.58)	8.88 (.91)	8.46 (.68)	ADHD > autism<br>ADHD > controls
Hyperactivity-impulsivity	59.17	< .001	.39	21.07 (.65)	12.51 (1.01)	10.94 (.76)	ADHD > autism<br>ADHD > controls
Routine	29.18	< .001	.24	9.93 (.25)	13.32 (.39)	10.12 (.29)	Autism > ADHD<br>Autism > controls
Switching	22.56	< .001	.20	11.38 (.25)	12.98 (.40)	9.74 (.30)	Autism > ADHD<br>Autism > controls<br>ADHD > controls
Numbers/patterns	11.24	< .001	.11	9.73 (.40)	12.94 (.63)	9.53 (.47)	Autism > ADHD<br>Autism > controls
Social skills	45.00	< .001	.33	15.19 (.41)	22.11 (.65)	15.51 (.48)	Autism > ADHD<br>Autism > controls
Imagination	30.72	< .001	.25	13.30 (.42)	19.27 (.66)	14.06 (.49)	Autism > ADHD<br>Autism > controls

Note. * EMM = Estimated Marginal Mean, ** SE = Standard Error.

Table S4. Overview of Missing Data Across Study Variables

**Variable**	**N valid**	**N missing**	**% Missing**
Diagnostic group	199	0	0.0
PMDD provisional diagnosis (PSST)	199	0	0.0
PMS provisional diagnosis (PSST)	199	0	0.0
ADHD-RS Inattention	189	10	5.0
ADHD-RS Hyperactivity/Impulsivity	189	10	5.0
AQ-28 total score	192	7	3.5
AQ-28 Routine	192	7	3.5
AQ-28 Switching	192	7	3.5
AQ-28 Numbers/Patterns	192	7	3.5
AQ-28 Social Skills	192	7	3.5
AQ-28 Imagination	192	7	3.5
GSQ-NL Hypersensitivity (Sample A only)	162	3	1.8

Note. PSST = Premenstrual Symptoms Screening Tool; ADHD-RS = ADHD Rating Scale; AQ-28 = Autism-Spectrum Quotient short; GSQ-NL = Dutch Glasgow Sensory Questionnaire. The GSQ-NL was administered only in Sample A (n = 180), explaining the higher proportion of missing data for this measure.

Table S5. Means, Standard Deviations, and Independent-Samples *t*-Test Results by University

	**RU (n = 31)**		**UvA (n = 37–39)**				
	M	*SD*	*M*	*SD*	***t***	***df***	***p***
ADHD attention symptoms	6.81	5.29	8.65	5.92	−1.34	66	.185
ADHD hyperactivity-impulsivity symptoms	10.13	5.70	10.68	6.81	−0.36	66	.724
ADHD total	16.94	10.42	19.32	11.84	−0.88	66	.385
AQ routine	9.13	1.52	10.51	2.49	−2.81[a]	60.76	.007
AQ switching	8.77	1.93	10.03	2.59	−2.23	66	.029
AQ numbers/patterns	8.84	3.19	9.84	3.56	−1.21	66	.231
AQ social skills	13.26	2.87	16.14	4.99	−2.97[a]	59.05	.004
AQ imagination	13.13	2.87	14.54	4.13	−1.65[a]	63.96	.103
AQ total	53.13	7.42	61.05	10.82	−3.57[a]	63.68	<.001
GSQ total	—	—	46.56	13.88	—	—	—

Note. All *p*-values are two-tailed. RU = Radboud University; UvA = University of Amsterdam; AQ = Autism-Spectrum Quotient; GSQ data were unavailable for RU; no *t*-test was computed.
[a]Welch's *t*-test applied (Levene's test significant, $p < .05$).
